# Monitoring the Effectiveness and Safety of ARNI vs. Angiotensin Receptor Blocker by Frailty Status

**DOI:** 10.1093/geroni/igab046.802

**Published:** 2021-12-17

**Authors:** Dae Kim, Elisabetta Patorno, Chandrasekar Gopalakrishnan

**Affiliations:** 1 Hebrew SeniorLife, Boston, Massachusetts, United States; 2 Brigham and Women's Hospital and Harvard Medical School, Boston, Massachusetts, United States

## Abstract

Using Medicare data 2015-2017, we conducted 5 sequential 1-to-1 propensity score-matched analyses of ARNI initiators and angiotensin receptor blockers (ARB) initiators, mimicking the accrual of new data every 6 months. Primary effectiveness endpoint was a composite of heart failure hospitalization or all-cause mortality and primary safety endpoint was a composite of hospitalization or emergency department visits for hypotension, acute kidney injury, hyperkalemia, and angioedema. Among non-frail patients (n=5,014), the rates (per 100 person-years) for ARNI vs ARB were 12.7 and 9.2 (rate difference: 3.4, 95% CI: 0.8 to 6.1), respectively, for the effectiveness endpoint and 5.2 and 3.6 (rate difference: 1.5, 95% CI: -0.1 to 3.2), respectively, for the safety endpoint. Among frail patients (n=2,694), the corresponding rates were 19.8 and 21.6 (rate difference: -1.8, 95% CI: -7.0 to 3.4) for the effectiveness endpoint and 10.9 and 8.0 (rate difference: 2.9, 95% CI: -0.6 to 6.4) for the safety endpoint.

